# Acid-Suppressive Drugs and Risk of Fracture in Children and Young Adults: A Meta-Analysis of Observational Studies

**DOI:** 10.3389/fphar.2021.712939

**Published:** 2021-08-06

**Authors:** Jiangbi Li, Xiaoping Xie, Weibing Liu, Feng Gu, Ke Zhang, Zilong Su, Qiangqiang Wen, Zhenjiang Sui, Pengcheng Zhou, Tiecheng Yu

**Affiliations:** ^1^ Department of Orthopedics, The First Hospital of Jilin University, Changchun, China; ^2^ Department of Orthopedics, The First People’s Hospital of Yunnan Province, Kunming, China

**Keywords:** proton pump inhibitors, histamine-2 receptor antagonists, fracture, meta-analysis, medication

## Abstract

**Background:** Recent studies have suggested that proton pump inhibitors (PPIs) and histamine type 2 receptor antagonists (H2RAs) may increase the risk of fracture. We performed a meta-analysis to evaluate the risk of fracture with PPIs and H2RAs use in children and young adults.

**Methods:** PubMed, EMBASE database, Cochrane Library, and Web of Science for relevant articles published before May 2021 were searched. We included all the observational studies reporting on the risk of fracture with acid-suppressive drug (PPIs and H2RAs) use in children and young adults. We calculated pooled risk ratios (RRs) for fracture using random-effects models and conducted subgroup analyses.

**Results:** A total of six studies were included in our analysis. Pooled analysis of PPIs use showed significant risk for fracture (RR = 1.23; 95% CI, 1.12–1.34; *I* ^2^ = 79.3), but not significant for PPIs combined with H2RAs use (RR = 1.22; 95% CI, 0.94–1.60; *I* ^2^ = 44.0%), as well as for H2RAs use alone (RR = 1.08; 95% CI, 0.94-1.24; *I* ^2^ = 84.1%). Grouping of studies by region showed a significantly increased fracture risk with PPIs use in North America (RR = 1.24; 95% CI, 1.16–1.32; *I* ^2^ =0.0%) than in Europe (RR = 1.23; 95% CI, 1.00–1.52; *I* ^2^ = 94.6%) and Asia (RR = 1.10; 95% CI, 0.96–1.25). However, there was no significant association between the H2RAs use and the fracture risk in North America (RR = 1.08; 95% CI, 1.00–1.09; *I* ^2^ = 0.0%). Moreover, PPIs use showed an increased risk of fracture in women (RR = 1.13; 95% CI, 1.07–1.19; *I* ^2^ = 0.0%), whereas there was no significant association between the PPIs use and the risk of fracture in men (RR = 0.93; 95% CI, 0.66–1.31; *I* ^2^ = 0.0%).

**Conclusion:** PPIs use alone could increase the risk of fracture in children and young adults, but not for PPIs combined with H2RAs use or H2RAs use alone. Clinicians should exercise caution when prescribing PPIs for patients.

## Introduction

The use of PPIs and H2RAs among children has substantially increased in recent years (Hales et al., 2018). PPIs and H2RAs have shown to be effective drugs in children as well as in adults with acid-related gastrointestinal disorders, such as *Helicobacter pylori* infection and gastroesophageal reflux disease (GERD) (Herszényi et al., 2020; Perry et al., 2020). These drugs are generally considered safe, but children are more vulnerable to drug toxicity (Ward and Kearns, 2013). Accordingly, it is critical to clarify the safety of PPIs or H2RAs in children.

In adult studies, PPIs use can significantly increase the risk of fracture, particularly fractures of the spine and hip (Eom et al., 2011a; Kwok et al., 2011; Ngamruengphong et al., 2011). Recent studies have suggested that PPIs may also increase the risk of fracture in children and young adults; there are several biological mechanisms that explain a possible relationship between the PPIs use and the risk of fracture. Some studies found that PPIs can reduce calcium absorption through the inhibition of gastric acid secretion, which leads to increase of the risk of bone fracture (Yang et al., 2006; O’Connell et al., 2005). PPIs may decrease the absorption of vitamin B12 (Lewis et al., 2014), which is associated with bone mineral content and density in elderly women (Dhonukshe-Rutten et al., 2003). Moreover, PPIs might have direct deleterious effects on osteoclast and osteoblast cells, which might result in a reduction in bone turnover (Costa-Rodrigues et al., 2013).

H2RAs are an alternative medication to treat GERD in children and young adults (Cohen et al., 2015). One study showed that H2RAs are safer for the treatment of GERD in children and adolescents (Ruigómez et al., 2017), and there was no significant risk of fracture with H2RAs use (Kwok et al., 2011). However, another study reported a high risk of fracture among young adults receiving H2RAs (Freedberg et al., 2015a). Whether the use of H2RAs or PPIs can significantly increase the risk of fracture is still much debated. A number of studies have investigated the association between the PPIs or H2RAs use and the risk of fracture in children and young adults, but showed inconsistent results. To our knowledge, there is no comprehensive meta-analysis of the related studies yet. Therefore, we conducted such meta-analysis to identify the association between PPIs and/or H2RAs use and the risk of fracture in children and young adults.

## Methods

The MOOSE guidelines were adhered for meta-analysis of observational studies and PRISMA guidelines (Stroup et al., 2000).

### Literature Search Strategy

PubMed, EMBASE database, Cochrane Library, and Web of Science for relevant articles published before May 2021 were searched by two independent reviewers (Li and Xie). We used the following search terms: “proton pump inhibitors” OR “histamine type 2-receptor antagonists” AND “fracture, bone” OR “broken bones” AND “children” OR “young” OR “young adult” OR “infant” OR “adolescent.”

### Selection Criteria

Eligible studies were included if they fulfilled the following criteria: (1) cohort or case control studies, (2) reported on the risk of fracture with PPIs or H2RAs use, (3) the reference group were non-PPIs or -H2RAs users, and (4) studies provided adequate data for the risk estimates. The criteria for exclusion were as follows: (1) studies that only reported on bone density changes, (2) duplicated articles, (3) case reports, reviews, meta-analyses, editorials, and letters, (4) insufficient data, and (5) studies conducted in animals or cell lines.

### Data Extraction and Quality Assessment

The following data were extracted from each study: name of the first author, publication year, study design, country, sample size, age, sex proportion, study period, total participant, duration of follow-up, and effect sizes (risk ratios (RRs), odds ratios (ORs), and hazard ratios (HRs)). The quality of included studies was evaluated using the Newcastle–Ottawa Scale (NOS) (Stang, 2010), wherein a maximum score of nine reflects high quality and an NOS score of seven and eight indicate medium quality (Asbridge et al., 2012). Two authors (Li and Xie) independently extracted the data and assessed the quality of the included studies.

### Statistical Analysis

Analyses were performed using the Stata 12.0 software package. Because the incidence of fracture is sufficiently rare (<5% per year), ORs were considered as approximates of RRs. We extracted HRs, ORs, and RRs from the included studies; otherwise, we calculated unadjusted risk ratio using the raw data. The pooled risk ratio (RR) with 95% confidence intervals (CIs) from HRs and ORs was calculated using a random-effects model. The *I* ^2^ statistic and Q statistic were used to assess the heterogeneity across the included studies. *I* ^2^ > 50% and *p* < 0.05 indicated significant heterogeneity across studies. We performed a sensitivity analysis by removing one study per time to test the robustness of the results. The publication bias of the included studies was examined by the Begg funnel plot and Egger test.

## Results

**FIGURE 1:**
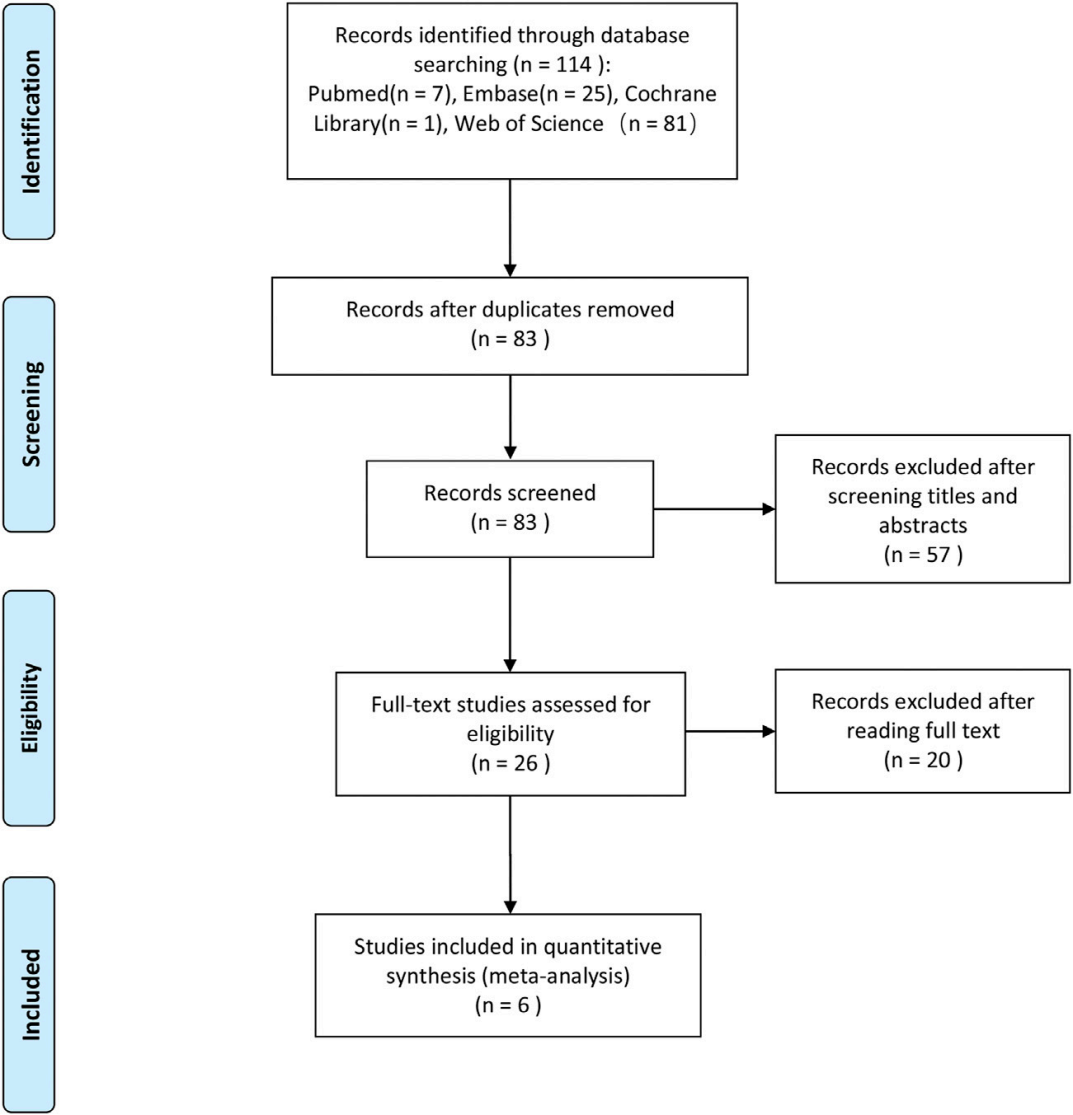
Flow diagram of the literature search process and study inclusion.

We initially identified 114 potentially eligible studies after the literature search process, and after removing 31 duplicated ones, 83 studies remained, in which we excluded 57 other studies which were obviously irrelevant based on the titles and abstracts. Also, after full-text assessment, we eventually identified six studies that could be used for our meta-analysis. The literature search process is illustrated in Figure 1.

### Study Characteristics

Six studies (Freedberg et al., 2015a; Fedida et al., 2019; Malchodi et al., 2019; Wagner et al., 2019; Fleishman et al., 2020; Wang et al., 2020) were included in our meta-analysis following the abovementioned exclusion and selection procedure. The main characteristics of the included studies are summarized in Table 1. They were published between 2015 and 2020 including five cohort studies (Fedida et al., 2019; Malchodi et al., 2019; Wagner et al., 2019; Fleishman et al., 2020; Wang et al., 2020) and one case-control study (Freedberg et al., 2015a). Three studies were conducted in North America (Malchodi et al., 2019; Wagner et al., 2019; Fleishman et al., 2020), two in Europe (Freedberg et al., 2015a; Wang et al., 2020), and one in Israel (Fedida et al., 2019). Six studies evaluated PPIs only; four evaluated H2RAs only; and three studies evaluated both PPIs and H2RAs. As shown in Table 1, the scores measured through the NOS ranged from six to nine points, suggesting that all the selected observational studies showed a reasonably good quality.

**TABLE 1:** Characteristics of the six included studies.

Study	Study design	Country	Age (years)	Sample size	% women	Study period	Agent: exposed/unexposed	Follow-up (years)	NOS quality score
Wang (2020)	Cohort	Sweden	<18	231866	51	2006–2016	PPIs: 115933/115933	5	9
Fedida (2019)	Retrospective cohort	Israel	18–25	488935	52	2007–2017	PPIs: 998/486372; H2RAs: 1347/486372; PPIs and H2RAs: 218/486372	2	6
Malchodi (2019)	Retrospective cohort	United States	3.6–9.1	851631	49	2001–2003	PPIs: 7998/754345; H2RAs: 71578/754345; PPIs and H2RAs: 17710/754345	2	9
Freedberg (2015)	Case control	United Kingdom	4–29	730442	35	1994–2013	PPIs: 2905/725854; H2RAs: 1591/725854; PPIs and H2RAs: 92/725854	≥1	6
Wagner (2019)	Retrospective cohort	United States	≥5	65432	49	2009–2010	PPIs; H2RAs^a^	≥5	8
Fleishman (2020)	Cohort	United States	0.5–15.5	64002	48	2011–2015	PPIs: 32001/32001	2	8

PPIs: proton pump inhibitors; H2RAs: histamine type 2 receptor antagonists.

a The original text does not provide exposed and unexposed data, only provide the OR value.

### Main Analysis

**FIGURE 2:**
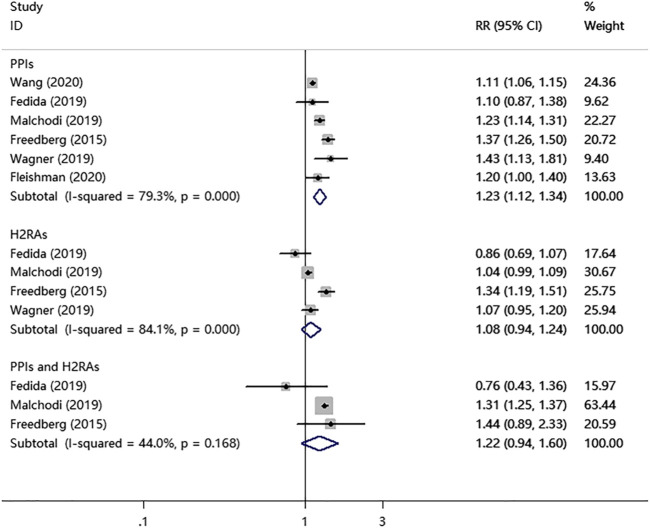
PPIs, H2RAs, or PPIs combined with H2RAs use and the combined risk of fracture in a random-effects model meta-analysis.

As shown in Figure 2, the PPIs use significantly increased the risk of fracture in children and young adults (RR = 1.23; 95% CI, 1.12–1.34; *I* ^2^ = 79.3%). In contrast, the H2RAs use did not show a significant association with the fracture risk (RR = 1.08; 95% CI, 0.94–1.24; *I* ^2^ = 84.1%). Interestingly, PPIs combined with H2RAs use also didn't increase the risk of fracture (RR = 1.22; 95% CI, 0.94–1.60; *I* ^2^ = 44.0%).

### Subgroup Meta-Analyses

Table 2 shows the risk of fracture in the subgroup meta-analyses. When the selected studies were grouped by type, we found a significant association between the PPIs use and the risk of fracture in all types. However, a positive association between the use of H2RAs alone and fracture risk was seen only in one case-control study (RR = 1.37; 95% CI, 1.26–1.50), and no significant association was seen in the three cohort studies (RR = 1.03; 95% CI, 0.95–0.11; *I* ^2^ = 35.2%).

**TABLE 2:** PPIs, H2RAs, or PPIs combined with H2RAs use and the risk of fracture in subgroup meta-analyses using a random-effects model.

Factor	No. of studies	RR (95% CI)	Heterogeneity test		Factor	No. of studies	RR (95% CI)	Heterogeneity test	
			*I*^2^, %	*p* Value				*I*^2^, %	*p* Value
**Study design**		**NOS score**				
**PPIs**	6	1.23 (1.12–1.34)	79.3%	0.000	**PPIs**	6	1.23 (1.12–1.34)	79.3%	0.000
Cohort	5	1.18 (1.01–1.28)	60.5%	0.038	High≥(7 point)	4	1.20 (1.10–1.31)	70.0%	0.018
Case control	1	1.370 (1.26–1.50)	-	-	Low<(7 point)	2	1.26 (1.02–1.55)	67.1%	0.081
**H2RAs**	4	1.08 (0.94–1.24)	84.1%	0.000	**H2RAs**	4	1.08 (0.94–1.24)	84.1%	0.000
Cohort	3	1.03 (0.95–0.11)	35.2%	0.214	High≥(7 point)	2	1.04 (1.00–1.09)	**0.0%**	0.659
Case control	1	1.370 (1.26–1.50)	-	-	Low<(7 point)	2	1.08 (0.70–1.67)	91.8%	0.000
**PPIs and H2RAs**	3	1.22 (0.94–1.60)	44.0%	0.168	**PPIs and H2RAs**	3	1.22 (0.94–1.60)	44.0%	0.168
Cohort	2	1.08 (0.65–1.80)	70.7%	0.065	High≥(7 point)	1	1.31 (1.25–1.37)	—	—
Case control	1	1.44 (0.89–2.33)	-	-	Low<(7 point)	2	1.07 (0.57–1.99)	64.1%	0.095
**Region**					**Outcome**				
**PPIs**	6	1.23 (1.12–1.3)	79.3%	0.000	**PPIs**	—	—	—	—
North America	3	1.24 (1.16–1.32)	**0.0%**	0.448	Spine	2	1.77 (0.86–3.63)	69.7%	0.069
Europe	2	1.23 (1.00–1.52)	94.6%	0.000	Upper limb	2	1.08 (1.04–1.13)	**0.0%**	0.590
Asia	1	1.10 (0.96–1.25)	-	-	Low limb	2	1.24 (1.08,1.43)	42.2%	0.188
**H2RAs**	4	1.08 (0.94–1.24)	84.1%	0.000	Other fracture	2	1.6 (1.26–2.02)	**0.0%**	0.352
North America	2	1.04 (1.00–1.09)	**0.0%**	0.659	**Sample size**	—	—	—	—
Europe	1	1.34 (1.19–1.51)	-	-	**PPIs**	—	—	—	—
Asia	1	0.86 (0.69–1.07)	-	-	>100000	4	1.20 (1.08–1.33)	86.4%	0.000
**PPIs and H2RAs**	3	1.22 (0.94–1.60)	44.0%	0.168	<100000	2	1.28 (1.09–1.52)	29.1%	0.235
North America	1	1.31 (1.25–1.37)	-	-	**Sex (PPIs)**	—	—	—	—
Europe	1	1.44 (0.89–2.33)	-	-	Men	2	0.93 (0.66–1.31)	83.8%	0.013
Asia	1	0.76 (0.43–1.36)	-	-	Women	2	1.13 (1.07–1.19)	**0.0%**	0.825

PPIs: proton pump inhibitors; H2RAs: histamine type 2 receptor antagonists; -: heterogeneity cannot be calculated from a single article.

When we grouped studies by region, we observed that the risk of fracture in patients with the PPIs use was higher in North America (RR = 1.24; 95% CI, 1.16–1.32; *I* ^2^ = 0.0%) and Europe (RR = 1.23; 95% CI, 1.00–1.52; *I* ^2^ = 94.6%) than that in Asia (RR = 1.10; 95% CI, 0.96–1.25). There was no significant association between the H2RAs use and the fracture risk in North America (RR = 1.08; 95% CI, 1.00–1.09; *I* ^2^ = 0.0%); however, the H2RAs use significantly increased the risk of fracture in Europe (RR = 1.34; 95% CI, 1.19–1.51). There was also a significant positive association between the combined PPIs and H2RAs use and the fracture risk in North America (RR = 1.31; 95% CI, 1.25–1.37).

Grouping of studies by NOS score showed a significant positive association between the PPIs use and the fracture risk in both the high-quality studies (RR = 1.20; 95% CI, 1.10–1.31; *I* ^2^ = 70.0%) and low-quality ones (RR = 1.26; 95% CI, 1.02–1.55; *I* ^2^ = 67.1%). In contrast, we found no association between the H2RAs use and the fracture risk in both the high-quality studies (RR = 1.04; 95% CI, 1.00–1.09; *I* ^2^ = 0.0%) and low-quality ones (RR = 1.08; 95% CI, 0.70–1.67; *I* ^2^ = 91.8%). Moreover, H2RAs combined with PPIs use has a significant association with the fracture risk in the high-quality studies (RR = 1.31; 95% CI, 1.25–1.37), but not in the low-quality ones (RR = 1.07; 95% CI, 0.57–1.99; *I* ^2^ = 64.1%).

When we grouped studies by sample size, we found a significant association between the PPIs use and the fracture risk in both the studies with more than 100,000 participants included (RR = 1.20; 95% CI, 1.08–1.33; *I* ^2^ = 86.4%) and those with less than 100,000 participants included (RR = 1.28; 95% CI, 1.09–1.52; *I* ^2^ = 29.1%).

In the subgroup meta-analyses by fracture outcome, the PPIs use increased the risk of low limb fracture (RR = 1.24; 95% CI, 1.08–1.43; *I* ^2^ = 42.2%), and other fracture (RR = 1.6; 95% CI, 1.26–2.02; *I* ^2^ = 0.0%).

The subgroup analyses by sex showed a significant association between the PPIs use and the risk of fracture in women (RR = 1.13; 95% CI, 1.07–1.19; *I* ^2^ = 0.0%), whereas there was no significant association between the PPIs use and the risk of fracture in men (RR = 0.93; 95% CI, 0.66–1.31; *I* ^2^ = 0.0%).

### Sensitivity Analysis and Publication Bias

The results of the sensitivity analysis demonstrated the stability of outcomes in meta-analyses. Begg’s test and Egger’s test were conducted, and there was no evidence for publication bias detected in all the analyses.

## Discussion

In this meta-analysis concerning the six selected observational studies, we found an increased risk of fracture with the PPIs use alone or the PPIs combined with H2RAs use in children and young adults, but not with the H2RAs use alone, which is consistent with the meta-analysis results of the acid-suppressing medication and fracture risk in adults (Kwok et al., 2011; Freedberg et al., 2015a). Besides, the use of PPIs or H2RAs increased the risk of fracture more significantly in one case-control study as compared to the cohort studies. However, it is generally considered that cohort studies provide superior evidence compared with case-control studies because cohort studies are less susceptible to bias in the prospective design. As a result, the results obtained from this meta-analysis should be interpreted with caution. Interestingly, the subgroup analyses by sex showed that the PPIs use increased the risk of fracture in women, but not in men. The subgroup analyses by fracture outcome showed an increased risk of spine, low limb, and other fracture for the PPIs use. The region of the study conducted may be one of the sources of heterogeneity among the studies. After limiting to North America, there was low heterogeneity across the related studies (*I* ^2^ = 0.0%, *p* > 0.05).

PPIs use is becoming increasingly common, and there has been an observed increase in the risk of gastrointestinal and respiratory tract infection (Eom et al., 2011b; Freedberg et al., 2015b), hypomagnesaemia, vitamin B12 deficiency, and bone fracture (De Bruyne and Ito, 2018). Particularly, the use of PPIs or H2RAs might increase the risk of bone fracture and osteoporosis (Targownik et al., 2010; Liu et al., 2019). The risk of fracture with PPIs use was higher than that with H2RAs use, which might be explained by a few reasons. First, PPIs have a stronger inhibitory effect on the acid production than H2RAs; the latter only suppress 70% of gastric acid production (Colin-Jones, 1995), whereas the former, PPIs, block 98% of the acid production (Schuler, 2007). Insoluble calcium salt from diet is difficult to absorb (Insogna, 2009), and the ionized calcium from insoluble calcium salts is promoted to release in the acidic environment of the stomach; thus, the reduced acid production could impair calcium absorption (O’Connell et al., 2005). Second, it was also reported that calcium malabsorption would cause hyperparathyroidism, thus leading to the reduction of bone mineral density (Insogna, 2009). Third, PPIs use could reduce vitamin B12 absorption, which may increase the risk of fracture as well (Marcuard et al., 1994). Fourth, cimetidine, one of the H2RAs, has been shown to increase bone mineral density by preventing osteoclast differentiation induced by histamine (Yamaura et al., 2003). Similarly, Vesterdaard et al. (2006) found that PPIs use might increase the risk of fracture whereas the H2RAs use decreases the risk. Although PPIs use was associated with increased risk of fracture, some studies found that there was no significant difference between the PPIs use and the level of bone mineral density (Targownik et al., 2010; Targownik et al., 2012; Hansen et al., 2019). Last, PPIs might increase the incidence of nonosteoporotic fracture by increasing the risk of falling (Thaler et al., 2016).

Our meta-analysis has several strengths. First, there were several previous meta-analyses on the fracture risk with PPIs or H2RAs use in the elderly. However, the current meta-analysis is the first to review the PPIs use and fracture risk in children and young adults. Second, it examined the associations stratified by the type of agent (PPIs or H2RAs), the study design, region, NOS score, outcome, and sample size, as well as sex. However, our meta-analysis also has several limitations. First, there was no available RCT on the issue, so our study consisted entirely of observational studies, which are also susceptible to various biases. Second, the number of eligible studies selected in our meta-analysis was relatively small. Third, some potential studies might have been missed with the language of publications restricted to English only.

## Conclusion

In summary, this meta-analysis of observational studies showed that PPIs use alone could increase the risk of fracture in children and young adults, but not for PPIs combined with H2RAs use or H2RAs use alone. Clinicians should carefully assess the risk of fracture before prescribing PPIs for patients. Further studies are required to confirm our findings reported hereby due to the inevitable limitations of this meta-analysis.

## Data Availability

The raw data supporting the conclusions of this article will be made available by the authors, without undue reservation.
